# Effectiveness of the Human Papillomavirus Vaccine in Extended Age Groups: A Real-World Analysis Based on the Korean HPV Cohort Study

**DOI:** 10.3390/cancers17152561

**Published:** 2025-08-03

**Authors:** Heekyoung Song, Sanha Lee, Suein Choi, Soo Young Hur

**Affiliations:** 1 Department of Obstetrics and Gynecology, Incheon St. Mary’s Hospital, College of Medicine, The Catholic University of Korea, Seoul 21431, Republic of Korea; songdeng86@catholic.ac.kr; 2 Department of Obstetrics and Gynecology, Seoul St. Mary’s Hospital, College of Medicine, The Catholic University of Korea, Seoul 06591, Republic of Korea; sanha.lee0327@gmail.com; 3 Department of Pharmacology, College of Medicine, The Catholic University of Korea, Seoul 06591, Republic of Korea; 4 Division of Data Science, PIPET, College of Medicine, The Catholic University of Korea, Seoul 06591, Republic of Korea

**Keywords:** HPV vaccine, extended age, South Korea

## Abstract

Background/Objectives: This study evaluated the real-world effectiveness of prophylactic Human Papillomavirus (HPV) vaccination in Korean women aged over 26 years, focusing on its impact on persistent HPV infection and disease progression. Methods: This multicenter prospective study analyzed data from the Korea HPV Cohort (2010–2021). After applying exclusion criteria, the final analytical cohort included 1,231 women aged ≥ 27 years with cytologic findings of atypical squamous cells of undetermined significance/low-grade squamous intraepithelial lesions and HPV infection. Propensity score matching was used to compare vaccinated (*n* = 340) and unvaccinated (*n* = 891) participants. After matching, 273 vaccinated and 273 unvaccinated individuals were included in the final analysis. The primary outcomes were persistent HPV infection and progression to biopsy-confirmed cervical intraepithelial neoplasia grade 2 or worse (CIN2+). Logistic and Cox regression models were employed, with additional age-stratified analyses. Results: Among women aged 27–39 years, vaccination was significantly associated with a 54% reduction in the odds of persistent HPV infection (odds ratio = 0.46; 95% CI: 0.22–0.96; *p* = 0.040). In the full cohort, vaccinated participants had a 62% lower risk of progression to CIN2+ compared with unvaccinated participants (hazard ratio = 0.38; 95% CI: 0.18–0.81; *p* = 0.011). Body mass index had a notable impact on HPV persistence in HPV 16/18 genotype groups. Conclusions: HPV vaccination effectively reduced persistent infection and progression to CIN2+ in Korean women, particularly those vaccinated before age 40. These findings support the age-extended HPV vaccination policies in South Korea.

## Simple Summary

This study evaluated the clinical effectiveness of prophylactic HPV vaccination in Korean women aged 27 years and older with ASCUS or LSIL cytology. Using data from the Korea HPV Cohort (2010–2021), 1,231 HPV-positive women were analyzed, including 273 matched pairs of vaccinated and unvaccinated participants. Among women aged 27–39 years, vaccination significantly reduced the odds of persistent HPV infection by 54%. In the full cohort, vaccinated women had a 62% lower risk of progression to CIN2+ compared to unvaccinated women. While limited to women with ASCUS or LSILs, these findings provide evidence for potential clinical benefits of HPV vaccination in extended age groups and may help inform future vaccination strategies in Korea.

## 1. Introduction

Cervical cancer was the fourth most common cancer among women worldwide in 2022, with an estimated 660,000 new cases and 350,000 deaths [1]. In South Korea, the age-standardized incidence rate was 3.9 per 100,000 individuals in 2021 [2]. Although global trends vary, a significant decline in cervical cancer incidence has been observed in countries such as Sweden [3] and South Korea, where the rate dropped from 8.6 per 100,000 in 1999 to 3.9 per 100,000 in 2021 [2].

The global decline is largely attributed to the implementation of regular screening programs and the long-term effectiveness of prophylactic Human Papillomavirus (HPV) vaccination. In randomized controlled trials (RCTs) of bivalent and quadrivalent HPV vaccines, 11-year follow-up data showed no cases of HPV-related cancer among vaccinated adolescents, in contrast to the control group [4]. Additionally, multiple systematic reviews—including a Cochrane Review—have confirmed a significantly reduced incidence of cervical intraepithelial neoplasia grade 2 or worse (CIN2+) in vaccinated individuals aged 15–26 years compared with their unvaccinated counterparts [relative risk (RR), 0.01] [5,6].

Based on these findings, the United States Centers for Disease Control and Prevention recommends HPV vaccination for individuals aged 9–26 years: a two-dose schedule administered 6–12 months apart for those aged up to 14 years, and a three-dose schedule over a 6-month period for those aged 15–26 years [7]. In South Korea, HPV vaccination has been included in the national vaccination program since June 2016 for all girls aged 12–17 years, as well as for women aged 18–26 years from the second-lowest income bracket [8].

Although vaccine effectiveness is lower in individuals aged 26–45 years than in younger populations, a RR of 0.3 still indicates a meaningful risk reduction [5]. Recently, Korean guidelines have endorsed selective HPV vaccination for individuals up to 45 years of age [9], aligning with the recommendations of the Centers for Disease Control and Prevention [7,10]. However, due to limited evidence from studies specifically focused on the Korean population, researchers have sought to analyze HPV cohort data to evaluate vaccine efficacy in this extended age group. Building on this, the present study aimed to assess the real-world effectiveness of prophylactic HPV vaccination in Korean women aged over 26 years, focusing on its impact on persistent HPV infection and disease progression.

## 2. Materials and Methods

### 2.1. Data Source

This multicenter study, part of the Korea HPV Cohort Study supported by the Korea Disease Control and Prevention Agency, was conducted from April 2010 to September 2021 at eight general hospitals across Korea. The objective was to identify risk factors for cervical disease progression to high-grade squamous intraepithelial lesions (HSIL) in HPV-positive Korean women.

Eligible participants were women aged 20 years or older with cytological findings of either atypical squamous cells of undetermined significance (ASCUS) or low-grade squamous intraepithelial lesions (LSILs), and who tested positive for HPV DNA. All participants provided written informed consent. HPV DNA testing and cytology were performed every six months, and clinical data were systematically collected using electronic case report forms [11].

### 2.2. Ethical Statement

The study received approval from the Institutional Review Board (IRB) of all participating institutions, and subsequent analyses of the data were also approved by the IRB of Seoul St. Mary’s Hospital, College of Medicine, The Catholic University of Korea (IRB No. KC24ZNDI0342).

### 2.3. Cohorts

**Figure 1:**
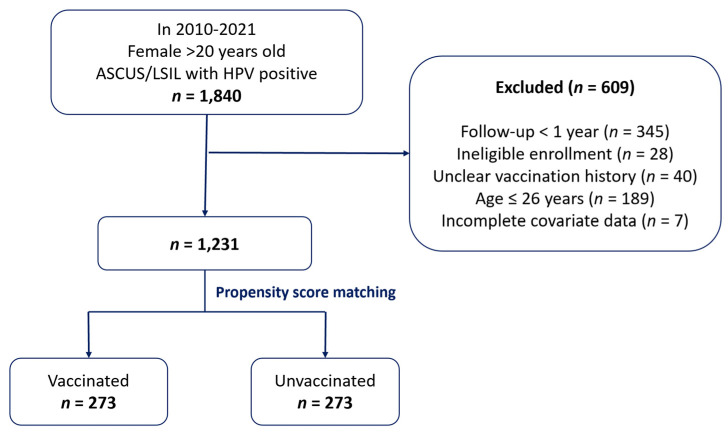
Flow diagram showing cohort selection, exclusion criteria, and 1:1 propensity score matching.

Among 1840 HPV-positive women aged ≥20 years with ASCUS or LSIL cytology enrolled between 2010 and 2021, 609 were excluded due to short follow-up (<1 year), enrollment ineligibility, unclear vaccination history, young age (≤26 years), or missing covariates. The final analytic cohort included 1,231 participants, of whom 546 (273 per group) were selected for matched analysis (Figure 1).

### 2.4. Study Population

Participants were categorized into vaccinated and unvaccinated cohorts based on HPV vaccination status. The unvaccinated group included women aged ≥27 years who remained unvaccinated throughout follow-up, while the vaccinated group comprised those who received the HPV vaccine at age ≥ 27 before study completion. For vaccinated participants, age at vaccination was determined using the most reliably available dose date—prioritizing the third dose, then the second, and finally the first. Those missing all three dates were excluded.

### 2.5. Study Outcomes and Covariates

The primary outcomes were HPV persistence and biopsy-confirmed disease progression. HPV-persistent infection was identified when the HPV test remained positive in two or more successive evaluations. The criterion for biopsy progression was established as a diagnosis of CIN2+, confirmed with biopsy [11]. Disease progression was defined as a biopsy-confirmed diagnosis of CIN2 or worse (CIN2+). For time-to-event analysis using the Cox model, progression was measured from enrollment to the first CIN2+ diagnosis. Study covariates were selected to adjust for potential confounding factors and included both continuous and categorical variables.

HPV genotyping was performed using a customized assay provided by Seegene Inc., Seoul, Korea, capable of detecting 57 HPV genotypes (Text S1). Although three vaccines—Cervarix, Gardasil 4, and Gardasil 9—were included, stratified analysis by vaccine type was not conducted due to the limited number of Gardasil 9 recipients (*n* = 1). Accordingly, HPV types were classified as HPV 16/18 versus non-16/18 types (e.g., HPV 31, 33, 35, 39, 45, 51, 52, 56, 58, 59, 66, and 68), based on vaccine coverage and genotype distribution within the study population. In cases of multiple HPV infections, the genotype with the highest oncogenic potential and longest duration of infection was selected as the representative type for analysis.

Continuous covariates included age at enrollment, height (cm), body weight (kg), and age at menarche. Categorical variables included sociodemographic characteristics (educational attainment, occupation, and monthly income); health-related behaviors (smoking status, alcohol consumption, and regular physical activity); reproductive history (menopausal status, pregnancy history, breastfeeding, and oral contraceptive use); comorbidities (hypertension and diabetes); and family history of malignancies (including cervical, breast, gastric, hepatic, endometrial, ovarian, and colorectal cancers). These variables were included to account for demographic, lifestyle, and clinical factors that may influence HPV outcomes and vaccine effectiveness.

### 2.6. Statistical Analysis

Prior to statistical analysis, propensity score matching (PSM) was conducted for participants aged ≥27 years (*n* = 1231) using nine covariates based on causal diagram analysis, including age, BMI, education, lifestyle, reproductive, and family history factors (Figure S1) [12]. Logistic regression estimated propensity scores with 1:1 nearest-neighbor matching (caliper = 0.1 SD of the logit). Post-matching balance was confirmed (standardized mean differences < 0.1). For unvaccinated participants, vaccination age was imputed using a CatBoost machine learning model (CatBoost; version 1.2.2, https://catboost.ai, accessed at 20 February 2025) excluding vaccination status as a predictor (see Supplementary Methods, Text S2).

Within the matched cohort, logistic regression was used to assess HPV persistence, and Cox proportional hazards models were applied to evaluate time-to-event progression. Both models included vaccination status, age at enrollment, BMI, and high-risk HPV status as covariates. Interaction terms were also evaluated to explore potential synergistic effects between vaccination and other risk factors. The evaluated covariates were selected based on a causal framework using Information Complexity (ICOMP) and their clinical relevance to both vaccination and outcomes (Figure S2) [13]. To account for limited event numbers, the Cox model included stratification by matched pair identifiers and used robust standard errors. Subgroup analyses with various age cutoffs were conducted to determine the optimal vaccination age range. For unvaccinated participants, enrollment age was used as a proxy for vaccination age. All analyses were based on complete cases (<1% missing data) and performed using R version 4.3.1 (R Foundation for Statistical Computing, Vienna, Austria, https://www.r-project.org/, accessed at 20 February 2025), with statistical significance defined as *p* < 0.05.

## 3. Results

### 3.1. Patient Characteristics

Baseline characteristics are presented in Table 1. A total of 1,231 participants were included, comprising 891 (72.38%) unvaccinated and 340 (27.62%) vaccinated individuals. Vaccinated participants were significantly younger at enrollment than unvaccinated participants (mean age 35.13 ± 6.50 vs. 44.19 ± 8.62 years; *p* < 0.001). Among the vaccinated group, the mean age at vaccination was 34.24 ± 6.45 years.

Significant differences were found between the groups in educational attainment, with a higher proportion of graduates or above among vaccinated participants (56.47% vs. 34.45%; *p* < 0.001). Reproductive health characteristics also significantly differed, with lower pregnancy rates (59.41% vs. 86.76%) and higher oral contraceptive use (22.06% vs. 13.69%) in the vaccinated group (both *p* < 0.001).

Smoking prevalence was higher among vaccinated participants (19.12% vs. 11.22%; *p* < 0.001). Significant between-group differences were also observed in anthropometric measurements, BMI distribution, alcohol consumption, and exercise habits (all *p* < 0.001). Family histories of malignancies did not significantly differ between groups (30.88% vs. 29.74%; *p* = 0.696).

After PSM, a total of 546 participants (273 in each group) were included in the matched cohort. No statistically significant differences remained between the vaccinated and unvaccinated groups across any baseline variable (Table S1).

### 3.2. HPV Persistence Outcome

Multivariate logistic regression analysis was performed on the propensity score-matched dataset to identify factors associated with HPV persistence, with results summarized in Table 2.

Age at enrollment was positively associated with persistence, with every 9.06-year increase corresponding to 67% higher odds (OR = 1.67, 95% CI: 1.28–2.20, *p* < 0.001). However, a significant interaction between age and vaccination status (OR = 0.61, 95% CI: 0.42–0.88, *p* = 0.009) indicated that the age-related risk increase was attenuated in vaccinated individuals.

BMI also showed notable associations. Underweight individuals had significantly lower odds of persistence than those with normal weight (OR = 0.47, 95% CI: 0.29–0.76, *p* = 0.002), while overweight status was not significant (OR = 0.77, 95% CI: 0.47–1.27, *p* = 0.303). Interestingly, HPV 16/18 infection was associated with lower persistence compared to non-HPV 16/18 types (OR = 0.66, 95% CI: 0.45–0.97, *p* = 0.033), but also a significant interaction emerged: underweight individuals infected with HPV 16/18 had markedly higher odds of persistence (OR = 3.83, 95% CI: 1.53–9.85, *p* = 0.005), suggesting a synergistic risk effect.

Vaccination status alone was not significantly associated with persistence in the overall analysis (OR = 0.84, 95% CI: 0.60–1.17, *p* = 0.297).

### 3.3. Subgroup Analysis

**Figure 2:**
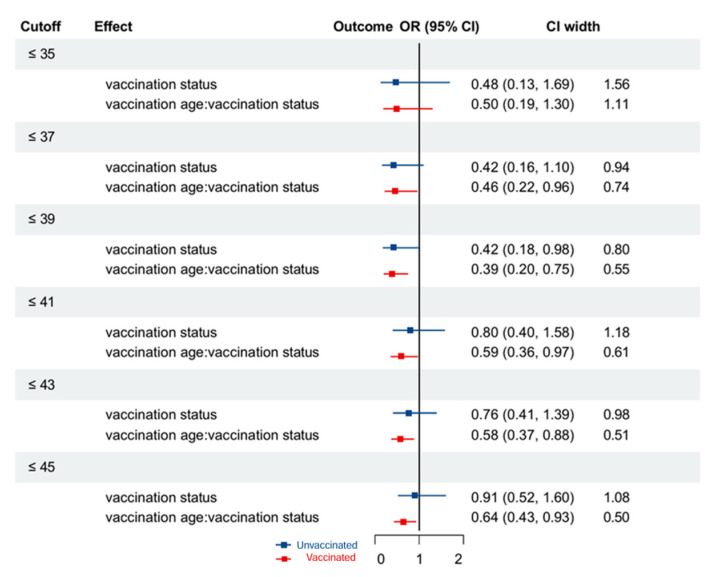
Forest plot of the odds ratios and 95% confidence intervals for each cutoff point determined by logistic regression analysis.

To investigate age-dependent vaccination effectiveness, we systematically evaluated optimal age cutoffs for cohort stratification by examining the main effects of vaccination and age-vaccination interactions in 2-year increments from 35 to 45 years. Vaccination status did not show a significant effect on HPV persistence in the overall analysis. However, in the <40 years subgroup, vaccination status had a significant protective effect, with vaccinated individuals being 0.46 times less likely to have persistent HPV infection (95% CI: 0.22−0.96, *p* = 0.040). The 39-year threshold (OR = 0.42, 95% CI = 0.80) demonstrated the strongest effect, while beyond age 41, CIs widened substantially (e.g., OR = 0.80, 95% CI = 1.18), reflecting increased uncertainty. Therefore, age 39 was selected as the primary cutoff point for subsequent subgroup analyses (Figure 2). Detailed results of the subgroup analysis are presented in Table 3.

A subgroup analysis among vaccinated participants aged ≤39 was conducted; results are presented in Table 4. Among vaccinated participants, vaccination age did not significantly influence HPV persistence (OR = 0.72, 95% CI: 0.44–1.17, *p* = 0.186). However, a significant interaction between vaccination age and HPV risk classification was identified (interaction OR = 15.55, 95% CI: 3.68–83.48, *p* < 0.001). This indicates that among participants infected with HPV 16/18, a higher vaccination age was associated with higher odds of viral persistence compared to those without HPV 16/18 infection.

### 3.4. Cox Proportional Hazards Analysis

**Figure 3:**
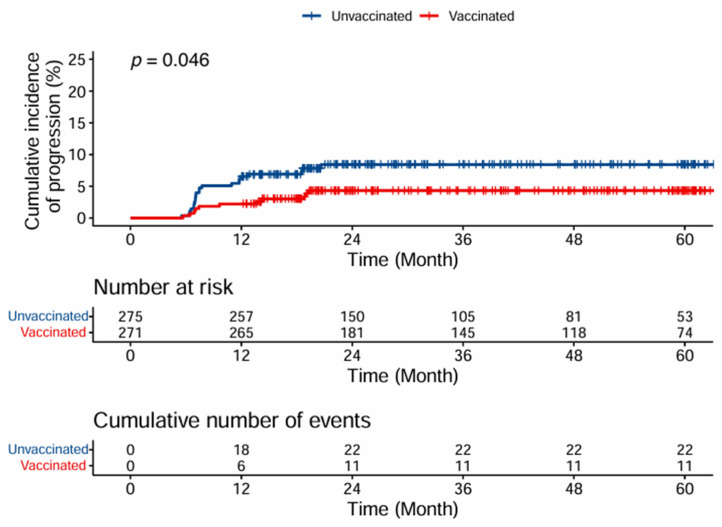
Cumulative incidence of biopsy progression over time by vaccination status.

Cox proportional hazards regression was performed on the propensity score- matched cohort to examine time-to-biopsy progression. Two participants who were vaccinated after the last biopsy were reclassified as unvaccinated for this analysis, resulting in 275 unvaccinated and 271 vaccinated participants. Cumulative incidence analysis demonstrated lower progression rates in the vaccinated group, with 11 events versus 22 events in the unvaccinated group over 5 years (log-rank test *p* = 0.046) (Figure 3).

Vaccination demonstrated significant protective effects, with a hazard ratio of 0.38 (95% CI: 0.18–0.80, p = 0.011) for biopsy progression. HPV 16/18 infection status showed a non-significant trend toward increased progression risk (*p* = 0.094), suggesting potential clinical relevance despite not reaching conventional statistical significance. Also, BMI did not show any significant effect on biopsy progression (*p* = 0.227) (Table 5).

## 4. Discussion

This study evaluated the efficacy of prophylactic HPV vaccination in extended age groups of Korean women, particularly those with LSILs and ASCUS. Persistent infection with oncogenic HPV types is strongly associated with an increased risk of precancerous lesions and cervical cancer [14,15], highlighting the importance of effective vaccination strategies for this population.

To assess the impact of vaccination, we examined its effect on persistent HPV infection and progression to biopsy-confirmed CIN2+. Although this study was based on a cohort of women with LSILs and ASCUS rather than a population-based sample, it offers clinically relevant insights given the lack of prospective data on extended-age HPV vaccination in Korea. Because cytologic status at the time of vaccination was unknown, some misclassification may have attenuated the observed effectiveness. To address baseline differences, we employed PMS to minimize bias and improve the reliability of our findings.

Among individuals aged <40 years, HPV vaccination was associated with significantly lower odds of persistent infection compared to no vaccination (OR = 0.46). Similarly, a recent prospective cohort study conducted in India reported a reduction in persistent HPV infections following vaccination [16]. Previous Korean studies also demonstrated that vaccination significantly reduced persistent infections with HPV types 16/18 [17]; however, they did not assess age-specific effects.

While global guidelines currently recommend HPV vaccination up to age 45 [18], our findings suggest that targeting women under 40 may be a feasible and evidence-based strategy for Korean women. The reduced vaccine effectiveness observed with increasing age may be partially due to declining immunogenicity in older individuals and a greater likelihood of prior HPV before vaccination [19].

Notably, while current prophylactic HPV vaccines primarily target types 16/18, in our cohort, all but one vaccinated individual received either Gardasil 4 or Cervarix—both designed to protect against HPV types 16/18 [20,21]. These findings underscore the effectiveness of existing vaccines in targeting the intended genotypes.

In our analysis of HPV persistence revealed that participants infected with HPV 16/18 had lower odds of persistent infection compared to those with non-HPV 16/18. One possible explanation is the strong immunogenicity of prophylactic vaccines targeting HPV 16 and 18, which may lead to more efficient immune-mediated viral clearance in vaccinated individuals [20,21]. Supporting this, the odds ratio for persistence in the HPV 16/18 group increased to 1.05 among vaccinated individuals aged 27–39 years, although this difference did not reach statistical significance. Another plausible explanation is that it may reflect the distinct genotype distribution in Korea, where HPV types in the 50s—such as HPV 58 or 52—are relatively common and may contribute more significantly to persistent infections [22,23]. Additionally, partial cross-protection offered by HPV 16/18-targeted vaccines against non-vaccinated types, such as HPV 31 and 45, may have influenced this outcome [24,25]. Lastly our study defined persistence based on repeated HPV DNA detection, which may not fully reflect biologically active or transforming infections [26]. The observed discrepancy—lower persistence but higher risk of progression for HPV 16/18 in our Cox analysis—suggests that viral detection alone may not fully reflect oncogenic potential.

Furthermore, BMI was significantly associated with HPV persistence, particularly in its interaction with HPV risk type. Although underweight individuals exhibited reduced overall persistence, those infected with HPV types 16/18 had increased odds of persistent infection. These findings suggest that individuals with lower BMI may have impaired viral clearance due to reduced activation of the innate immune system, potentially resulting from deficiencies in essential omega-3 fatty acids—such as eicosatetraenoic acid and docosahexaenoic acid—as well as key vitamins and minerals [27]. This trend appears to be especially pronounced in infections with HPV 16/18. Conversely, obesity may also hinder viral clearance by reducing regulatory T cell populations, disrupting T helper cell balance, and impairing B cell function [28]. Therefore, both underweight and obese individuals may be at a disadvantage in clearing HPV infections. Although BMI was not associated with CIN2+ progression in our study, these findings underscore the potential need for targeted prevention strategies in metabolically vulnerable populations. Further research is warranted to clarify the underlying metabolic and immunological mechanisms involved in these associations.

Several cohort studies have evaluated the impact of HPV vaccination on pathological progression. A national cohort study conducted in Denmark reported a significant reduction in cervical cancer incidence among individuals vaccinated before 20 years of age. Specifically, the incidence rate ratio (IRR) was 0.14 (95% CI: 0.04–0.53) for those vaccinated at age 16 and 0.32 (95% CI: 0.08–1.28) for those vaccinated between ages 17 and 19 [29]. Similarly, a Swedish cohort study found that women vaccinated before age 17 had an IRR of 0.12 (95% CI: 0.00–0.34), whereas those vaccinated between ages 17 and 30 years had a higher IRR of 0.47 (95% CI: 0.27–0.75) [3].

Although our primary outcome was the incidence of CIN2+, our findings also align with prior studies by demonstrating a reduction in pathological progression across all vaccinated age groups. The cumulative incidence of progression was significantly lower in the vaccinated group than in the unvaccinated group (*p* = 0.046). Additionally, among individuals with ASCUS or LSILs, the HR for progression was 0.38 in the vaccinated group.

Furthermore, a similar trend emerged in the Cox regression analysis for disease progression: participants with HPV 16/18 infections showed a higher HR than those with other genotypes, although the difference was not statistically significant. These findings suggest that non-16/18 HPV types may play a more substantial role in the Korean population than previously recognized and warrant further investigation.

Although the specific age groups showing statistical significance differed from those identified in previous studies and in our own analysis of HPV persistence, the overall findings reinforce the protective effect of HPV vaccination in reducing the risk of pathological progression. To our knowledge, this is the first study to confirm the impact of HPV vaccination on disease progression using Korean cohort data. Furthermore, the results suggest that vaccination may offer protective benefits even for age groups beyond current guideline recommendations.

There are some limitations. First, although we assessed persistent HPV infection across different age groups, the subgroup analysis of pathological progression by age was limited by the small number of events. While a trend toward reduced CIN2+ incidence was observed in the vaccinated group, statistical significance was not achieved in the age-specific multivariate survival analysis for pathologic progression. This limitation calls for caution in interpreting age-specific progression effects and underscores the need for larger, age-stratified studies to validate these findings. Second, we did not directly assess the effectiveness of the nonavalent HPV vaccine (Gardasil 9), which covers a broader range of high-risk HPV types, including HPV 16, 18, 31, 33, 45, 52, and 58 [30]. Considering that HPV types in the 50s—particularly HPV 58—are relatively prevalent in the Korean population, evaluating the impact of the nonavalent HPV vaccine is especially important. However, due to the small number of individuals in our cohort who received the nonavalent vaccine, statistical analysis was not feasible. Although Gardasil 9 has been approved by the Korean Ministry of Food and Drug Safety (KFDA), it is not included in the National Immunization Program. As a result, its administration is limited, and due to the difficulty in tracking non-reimbursed prescriptions, prospective evaluation of its real-world effectiveness remains challenging. Finally, we were unable to conduct a formal cost-effectiveness analysis, which may limit the immediate applicability of our findings for public health decision-making. Although studies from the United States have reported that HPV vaccination up to age 45 may still offer marginal cost-effectiveness benefits under certain conditions [31,32], our study did not include an economic evaluation. Therefore, further research is war-ranted to assess the cost-effectiveness of extended-age HPV vaccination, particularly in the Korean healthcare setting.

Despite these limitations, this study leveraged prospective multicenter data with long-term follow-up to provide valuable real-world evidence on the effectiveness of HPV vaccination in South Korea. By evaluating both persistent HPV infection and pathological progression, this study offers a comprehensive assessment of vaccine impact. Furthermore, the use of PSM helped control for potential confounders, thereby strengthening the validity of the findings.

## 5. Conclusions

In summary, this study demonstrated that HPV vaccination reduced the persistence of infection in individuals under 40 years of age and lowered the risk of CIN2+ progression compared to the unvaccinated group. These findings support the potential extension of HPV vaccination policies to women aged over 26 years, particularly those under 40, and highlight the need for further studies on cost-effectiveness and feasibility.

## Figures and Tables

**Table 1:** Baseline demographics and clinical characteristics.

Variable	Cohort		*p*-Value ^a^
	Unvaccinated	Vaccinated	
	(*n* = 891) [Mean ± SD; n (%)]	(*n* = 340) [Mean ± SD; n (%)]	
Age at enrollment (y)	44.19 ± 8.62	35.13 ± 6.50	<0.001
Age at vaccination (y)	not applicable	34.24 ± 6.45	not applicable
Height (cm)	159.51 ± 5.22	161.72 ± 5.16	<0.001
Weight (kg)	56.59 ± 7.37	54.75 ± 7.65	<0.001
			<0.001
≤Normal weight	704 (79.01)	259 (76.18)	
Underweight	57 (6.40)	52 (15.29)	
Overweight	130 (14.59)	29 (8.53)	
Education level			<0.001
≤Middle school	124 (13.92)	6 (1.76)	
High school	321 (36.03)	54 (15.88)	
College	139 (15.60)	88 (25.88)	
University	243 (27.27)	150 (44.12)	
Graduate school	64 (7.18)	42 (12.35)	
Smoking			<0.001
Yes (five or more packs of cigarettes in entire lifetime)	100 (11.22)	65 (19.12)	
No	791 (88.78)	275 (80.88)	
Drinking			<0.001
Yes	583 (65.43)	281 (82.65)	
Past (1 year of sobriety since the last survey)	71 (7.97)	20 (5.88)	
None (never consumed alcohol in entire life)	237 (26.60)	39 (11.47)	
Regular exercise			<0.001
Yes (exercise regularly enough to sweat)	424 (47.59)	119 (35.00)	
No	467 (52.41)	221 (65.00)	
Pregnancy history			<0.001
Yes	773 (86.76)	202 (59.41)	
No	118 (13.24)	138 (40.59)	
Oral contraception use			<0.001
Yes	122 (13.69)	75 (22.06)	
No	769 (86.31)	265 (77.94)	
Family history of malignancy			0.696
Yes	265 (29.74)	265 (29.74)	
No	626 (70.26)	235 (69.12)	

^a^ Wilcoxon rank sum test; Pearson’s Chi-squared test. BMI, body mass index; SD, standard deviation.

**Table 2:** Multivariate logistic regression analysis results.

Variable	OR	95% CI	*p*-Value
Age	1.67	1.28, 2.20	<0.001
Vaccination status			0.297
Unvaccinated	*−*	*−*	
Vaccinated	0.84	0.60, 1.17	
HPV risk			0.033
Non-HPV 16/18	*−*	*−*	
HPV 16/18	0.66	0.45, 0.97	
BMI			
Normal weight	*−*	*−*	
Underweight	0.47	0.29, 0.76	0.006
Overweight	0.77	0.47, 1.27	
Age *×* Vaccination status			
Age *×* Vaccinated	0.61	0.42, 0.88	0.009
BMI *×* HPV risk			0.015
Underweight *×* HPV 16/18	3.83	1.53, 9.85	
Overweight *×* HPV 16/18	0.96	0.25, 3.53	

OR, odds ratio; CI, confidence interval; BMI, body mass index; HPV, Human Papillomavirus. “−” indicates the reference category; “×” denotes an interaction term.

**Table 3:** Multivariate logistic regression analysis results for the subgroup aged 27 to 39 years.

Variable	OR	95% CI	*p*-Value
Age	4.58	2.54, 8.50	0
Vaccination status			0.04
Unvaccinated	*−*	*−*	
Vaccinated	0.46	0.22, 0.96	
HPV risk			0.117
Non-HPV 16/18	*−*	*−*	
HPV 16/18	0.68	0.42, 1.10	
BMI			0.078
Normal weight	*−*	*−*	
Underweight	0.57	0.34, 0.94	
Overweight	1.08	0.60, 1.94	
Age *×* vaccination status			0.003
Age *×* Vaccinated	0.33	0.15, 0.68	

OR, odds ratio; CI, confidence interval; BMI, body mass index; HPV, Human Papillomavirus. “−” indicates the reference category; “×” denotes an interaction term.

**Table 4:** Multivariate logistic regression analysis results for the vaccinated subgroup aged 27 to 39 years.

Variable	OR	95% CI	*p*-Value
Vaccination age	0.72	0.44, 1.17	0.186
HPV risk			0.891
Non-HPV 16/18	*−*	*−*	
HPV 16/18	1.05	0.50, 2.29	
BMI			0.169
Normal weight	*−*	*−*	
Underweight	0.62	0.33, 1.13	
Overweight	0.6	0.27, 1.31	
Vaccination age *×* HPV risk			<0.001
Vaccination age *×* HPV 16/18	15.55	3.68, 83.48	

OR, odds ratio; CI, confidence interval; BMI, body mass index; HPV, Human Papillomavirus. “−” indicates the reference category; “×” denotes an interaction term.

**Table 5:** Cox proportional hazards regression analysis results.

Variable	HR	95% CI	*p*-Value
HPV risk			0.094
non-HPV 16/18	*−*	*−*	
HPV 16/18	2.39	0.86, 6.63	
BMI	0.54	0.20, 1.47	0.227
Vaccination status			0.011
unvaccinated	*−*	*−*	
vaccinated	0.38	0.18, 0.80	

HR, hazard ratio; CI, confidence interval; BMI, body mass index; HPV, Human Papillomavirus. “−” indicates the reference category.

## Data Availability

Individual participant data that underlie the results reported in this article, after de-identification, are available upon reasonable request from the corresponding author.

## Supplementary Materials

The following supporting information can be downloaded at: https://www.mdpi.com/article/10.3390/cancers17152561/s1, Figure S1: Causal diagram for variable relationships 1; Figure S2: Causal diagram for variable relationships 2; Table S1: Baseline demographics and clinical characteristics after PMS; Text S1: Full List of Detected HPV Genotypes; Text S2: Detailed Statistical analysis.

## Abbreviations

The following abbreviations are used in this manuscript:

ASCUS	Atypical squamous cells of undetermined significance
BMI	Body mass index
CIN2+	Cervical intraepithelial neoplasia grade 2 or worse
CI	Confidence interval
HR	Hazard ratio
HPV	Human Papillomavirus
HSIL	High-grade squamous intraepithelial lesions
IRB	Institutional review board
IRR	Incidence rate ratio
LSILs	Low-grade squamous intraepithelial lesions
OR	Odds ratio
PSM	Propensity score matching
RCT	Randomized controlled trial
RR	Relative risk
