# Supplementary material for: Effectiveness of the Human Papillomavirus Vaccine in Extended Age Groups: A Real-World Analysis Based on the Korean HPV Cohort Study
Source: Cancers (Basel). 2025 Aug 3;17(15):2561. doi: 10.3390/cancers17152561 (PMC12346625; doi:10.3390/cancers17152561)
Supplement: Supplementary file 1 [file cancers-17-02561-s001.zip › cancers-3775081-supplementary.pdf]

Supplementary

Figure S1: Causal diagram for variable relationships 1; Figure S2: Causal diagram for variable relationships 2; Table S1: Baseline demographics and clinical characteristics after PMS; Text S1: Full List of Detected HPV Genotypes; Text S2: Detailed Statistical analysis.

Text S1. Full List of Detected HPV Genotypes

This table presents all 57 HPV genotypes detected in this study, categorized according to oncogenic risk based on classifications by the International Agency for Research on Cancer (IARC) and relevant literature. Genotypes were grouped into three categories:

- **High-risk (HR):** Genotypes with confirmed carcinogenic potential, associated with cervical and other anogenital cancers (e.g., HPV 16, 18, 31, 33, 35, 39, 45, 51, 52, 56, 58, 59, 66, 68).
- **Possibly high-risk (pHR):** Genotypes with limited or inconclusive evidence regarding carcinogenicity (e.g., HPV 26, 30, 34, 53, 67, 69, 70, 73, 82, 97).
- **Low-risk (LR):** Genotypes considered non-oncogenic, primarily associated with benign conditions such as genital warts (e.g., HPV 1, 2, 3, 4, 6, 7, 8, 10, 11, 13, 22, 24, 32, 38, 40, 42, 43, 44, 54, 55, 57, 60, 61, 62, 63, 72, 81, 83, 84, 85, 90, 91).

Figure S1. Causal diagram for variable relationships 1

The causal diagram illustrates the direct relationships between key variables considered for inclusion in the analysis. Variables that formed direct paths were analyzed to identify those most relevant to the study's aims. Based on this analysis, variables such as "occupation",

“monthly average income”, and “breastfeeding” were excluded to avoid redundancy and bias in the matching process.

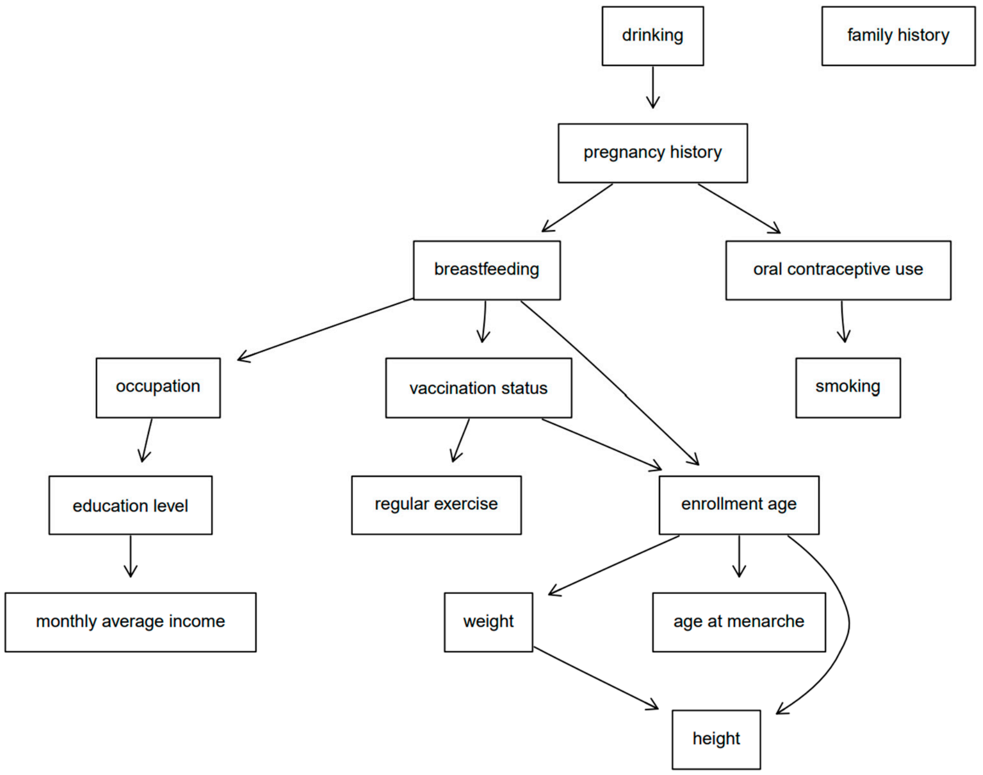

Text S2. Detailed Statistical analysis

1. Pre-processing of variables and missing data Prior to conducting the logistic regression analysis, several pre-processing steps were performed. These included redefining categorical variables, handling missing data, and standardizing continuous variables. For the redefinition of categorical variables, education level was simplified by cohorting individuals into three categories: “uneducated”, “elementary school”, and “middle school”. Similarly, occupation categories such as agricultural and fishing workers, function-related workers, and manual laborers were combined into one cohort. Monthly income categories were merged to create a manageable number of cohorts. The presence of missing values was identified in three

variables, however, the proportion of missing values in each variable was found to be less than 1%. Consequently, the decision was made to utilize complete case analysis. Continuous independent variables, such as “enrollment age”, “height”, “body weight”, and “age at menarche”, were standardized to facilitate comparison between cohorts and to improve the stability of the regression model. Matching for propensity score analysis (PSM) was performed using 1,231 observations, consisting only of those aged 27 and older.

2. Propensity Score Matching (PSM)

Before conducting PMS, several variables were removed to avoid potential biases and improve the matching process. First, variables with low response rates that could cause bias were excluded. The variable “menopausal” was removed because a majority of responses in the “Yes” category was from the unvaccinated cohort, which was found to be related to age. Similarly, variables such as “hypertension” and “diabetes” were removed due to their low response rates, which could lead to bias. Next, a causal diagram analysis was conducted to identify direct relationships between variables. The outcome variable was intentionally excluded from the diagram at this stage, as no covariates were found to have a direct or indirect causal relationship with the outcome. Accordingly, the diagram emphasizes the interrelationships among the covariates for the purpose of variable selection prior to propensity score matching. S1 Fig. displays this diagram, which shows the direct paths between variables, indicating their strong correlations. For instance, “education level”, “occupation”, and “monthly average income” were found to be closely related. Since “education level” alone could explain the variations among these variables, “occupation” and “monthly average income” were excluded. Similarly, “pregnancy history” and “breastfeeding” formed a direct path and were found to explain similar characteristics. Since

these variables were linearly related, “breastfeeding” was excluded.

Additionally, the variable “age at menarche” was excluded, as it may have been affected by recall bias, making it less reliable. Given its correlation with age, it was determined that its exclusion would not significantly affect the analysis. Furthermore, enrollment age, weight, and height were identified as interrelated variables. In order to address potential multicollinearity and more intuitively capture the complex relationship between height and weight, a derived variable, Body Mass Index (BMI), was calculated and used as a composite indicator. Given the relatively infrequent occurrence of obesity as defined by BMI criteria, the obesity category was combined with the overweight category. Consequently, the BMI variable was stratified into three categories: underweight, normal weight, and overweight. Consequently, BMI was the sole variable considered for propensity score matching and subsequent modeling processes, while height and weight were excluded to enhance the stability and interpretability of the analysis. After excluding six variables due to inadequate response rates and the absence of discernible causal relationships, and subsequently replacing height and weight with BMI, a total of nine variables were utilized in the PMS procedure to balance the covariates between the vaccinated and unvaccinated cohorts. The study incorporated a comprehensive set of nine variables, encompassing enrollment age, BMI, educational level, smoking habits, alcohol consumption, regular exercise routines, pregnancy history, oral contraceptive use, and family medical history. The caliper value was set to 0.1 times the standard deviation of the logit of the propensity score (PS) (=0.1×sd(logit(PS))), which was chosen to ensure proper matching while retaining a sufficient number of observations in the matched cohort. Although a caliper value of 0.2×sd(logit(PS)) is commonly used, this larger value resulted in insufficient balance for the key covariate vaccination age. Therefore, a caliper value of 0.1×sd(logit(PS)) was selected to improve match quality by better balancing vaccination age. As shown in S4 Table, this matching

strategy effectively improved covariate balance across the vaccinated and unvaccinated groups. The matching process successfully reduced imbalance in the PS, and after matching, all variables showed mean differences of less than 0.1, indicating well-balanced cohorts.

3. Causal Framework and Variable Selection

The initial variable selection was based on a causal diagram (S3 Fig.), which indicated that vaccination status and the outcome were independent, with no confounding path between them. Variables were selected to control for potential confounders and important risk factors for disease occurrence, based on the structure of the causal relationships. Vaccination status was incorporated into the analysis as the treatment variable, while enrollment age was designated as a confounder. This decision was informed by the understanding that enrollment age may be influenced by higher-level variables such as education level and regular exercise, and in turn, may affect both vaccination status and the incidence of disease. Although regular exercise was not included in the final model, it was considered to be partially represented by enrollment age in the causal structure. Moreover, since regular exercise is known to correlate with body composition, BMI was selected instead as a more clinically significant health indicator to adjust for physical health status. In addition, BMI, a composite measure of height and weight, was included as an independent predictor of disease outcome, despite its independence from vaccination status, due to its known association with various health outcomes including susceptibility to persistent HPV infection. Due to the potential for excessive complexity, the causal diagram was simplified to ensure interpretability. Although high-risk HPV types (e.g., types 16 and 18) are well-established risk factors for disease progression, incorporating them explicitly into the diagram would have made it overly complex and hindered clarity. Therefore, while these variables were omitted from the

diagram, they were included as adjustment variables in the statistical model due to their critical clinical relevance. Conversely, education level, pregnancy history, oral contraceptive use, and smoking were excluded from the final model, as their effects were deemed to be indirectly captured through the selected variables. Application of the Information Complexity Criterion (ICOMP) further supported model simplification: the exclusion of drinking and family history reduced model complexity while maintaining explanatory power. Consequently, the final model included vaccination status, enrollment age, BMI, and high-risk HPV status, thereby offering a concise yet effective framework for explaining HPV persistence. Interaction terms were also explored to assess whether the selected variables influenced the effect of vaccination on the outcome.

4. Performance Evaluation of the Vaccination Age Prediction Model for Imputation

To estimate the hypothetical vaccination age for unvaccinated individuals, a CatBoost regression model was trained using vaccinated individuals from the matched dataset. The predictive performance of the model was evaluated using a validation set consisting of vaccinated individuals who were not included in the matched data. Despite the absence of covariate-balancing with the unvaccinated group, this validation group facilitated a more conservative evaluation of the model's generalizability.

The CatBoost model exhibited the following predictive performance:

- Root Mean Squared Error (RMSE): 0.31
- Mean Absolute Error (MAE): 0.24
- Coefficient of Determination (R^2): 0.84

- Adjusted R^2: 0.81

Lower values of RMSE and MAE indicate smaller discrepancies between the predicted and actual vaccination ages, reflecting the model's accuracy in prediction. Specifically, the RMSE of 0.31 and the MAE of 0.24 suggest that the model's predictions deviate from the true values by only small amounts on average. The R^2 value of 0.84 indicates that approximately 84% of the variance in the actual vaccination age is explained by the model, signifying strong explanatory power. In general, R^2 values above 0.70 are considered indicative of good model performance. According to the metrics, the CatBoost model demonstrates notable predictive capability and is consistent with the estimated vaccination age in the unvaccinated population.

Figure S2. Causal diagram for variable relationships 2

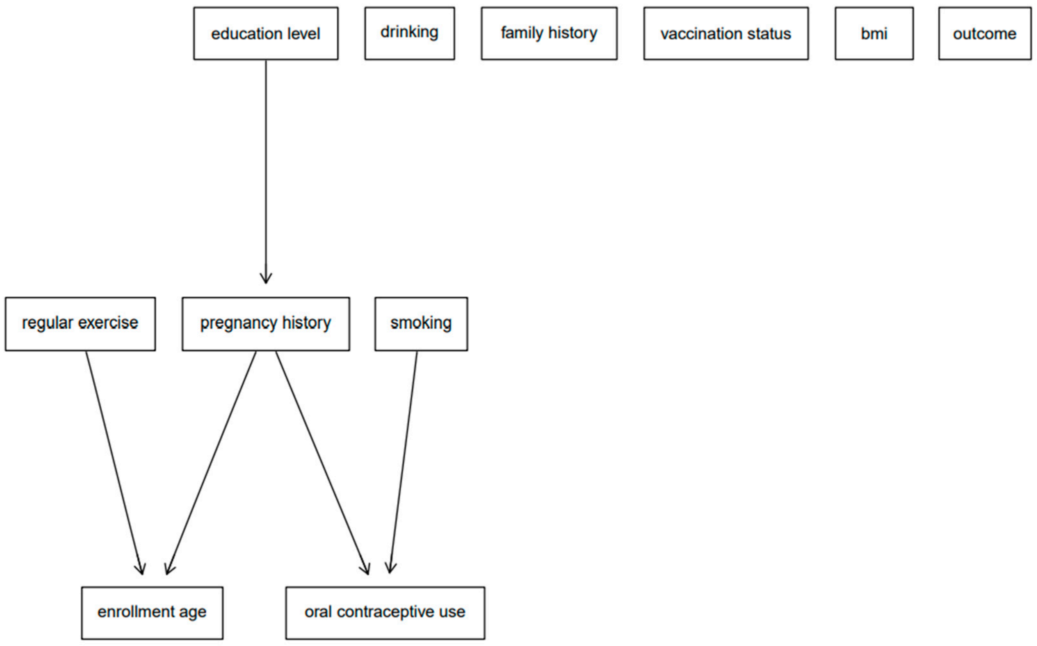

Table S1: Baseline demographics and clinical characteristics after PMS

Variables	Cohort		SMD[b]	p-value[c]
	**Unvaccinated** (*n* = 273[a])	**Vaccinated** (*n* = 273[a])		
**Age at enrollment (year)**	36.33 ± *6.62*	36.27 ± 6.75	0.01	*0.929*
**Age at vaccination (year)**	NA	35.38 ± 6.74	NA	NA
**Height (cm)**	161.34 ± 5.06	161.49 ± 5.20	0.03	*0.868*
**Weight (kg)**	55.37 ± 7.03	55.24 ± 7.85	0.02	*0.379*
**BMI**			0.09	*0.586*
≤ normal weight	216 (79.12)	206 (75.46)		
underweight	34 (12.45)	39 (14.29)		
overweight	23 (8.42)	28 (10.26)		
**Education level**			0.15	*0.548*
≤ middle school	2 (0.73)	6 (2.20)		
high school	57 (20.88)	52 (19.05)		
college	69 (25.27)	65 (23.81)		
university	118 (43.22)	116 (42.49)		
graduate school	27 (9.89)	34 (12.45)		
**Smoking**			< 0.001	> 0.999
yes (five or more packs of cigarettes in entire lifetime)	55 (20.15)	55 (20.15)		
no	218 (79.85)	218 (79.85)		
**Drinking**			0.1	0.511
yes	220 (80.59)	218 (79.85)		
past (one year of sobriety since last survey)	13 (4.76)	19 (6.96)		

none<br>(never consumed alcohol in entire life)	40 (14.65)	36 (13.91)		
**Regular exercise**			0.02	0.792
yes<br>(exercise regularly enough to sweat)	108 (39.56)	105 (38.46)		
no	165 (60.40)	168 (61.50)		
**Pregnancy history**			0.05	*0.53*
yes	181 (66.30)	174 (63.74)		
no	92 (33.70)	99 (36.26)		
**Oral contraception use**			0.11	0.184
yes	44 (16.12)	56 (20.51)		
no	229 (83.88)	217 (79.49)		
**Family history of malignancy**			0.13	0.122
yes	82 (30.04)	99 (36.26)		
no	191 (69.96)	174 (63.74)		

a) Mean ± SD; n (%)

b) Standardized Mean Difference

c) Wilcoxon rank sum test; Pearson's Chi-squared test

BMI, body mass index; NA, Not Applicable.
